# A Forensic Approach to Complex Identification Cases: The Collapse of an Italian Cemetery into the Sea

**DOI:** 10.3390/genes16030277

**Published:** 2025-02-25

**Authors:** Camilla Tettamanti, Francesca Frigiolini, Lorenzo Franceschetti, Rosario Barranco, Sara Lo Pinto, Lucia Casarino, Simonetta Verdiani, Mattia Porcu, Cristina Cattaneo, Danilo De Angelis, Marco Cummaudo, Francesco De Stefano, Francesco Ventura

**Affiliations:** 1 Department of Legal and Forensic Medicine, University of Genova, Via De Toni 12, 16132 Genova, Italy; camilla.tettamanti@unige.it (C.T.);; 2 Legal Medicine Unit, IRCCS-Ospedale Policlinico San Martino Teaching Hospital Genova, Largo Rosanna Benzi 10, 16132 Genova, Italy; 3 LABANOF, Laboratorio di Antropologia e Odontologia Forense, Institute of Legal Medicine, Department of Biomedical Sciences for Health, University of Milan, Via Luigi Mangiagalli 37, 20133 Milano, Italy

**Keywords:** forensic identification, disaster victim identification, cemetery collapse, human remains recovery, genetic profiling

## Abstract

Background/Objectives: On 22 February 2021, a coastal landslide in Italy caused the collapse of an old cemetery, displacing approximately 370 coffins, with over 200 plunging into the sea. This disaster necessitated the recovery and identification of human remains under challenging conditions to provide closure to families and uphold the dignity of the deceased. Methods: Recovery operations involved firefighters and scuba divers, followed by forensic analysis conducted by the Medical Staff of Legal and Forensic Medicine. A post-mortem team utilized forms adapted from Interpol’s Disaster Victim Identification (DVI) standards to document remains, which included 140 decomposed bodies and 193 bags of commingled skeletal remains. DNA samples were collected from 147 bone fragments, primarily long bones and teeth, and compared with ante-mortem data gathered from relatives. Results: Of the 77 eligible relatives, 66 consented to DNA sample collection for genetic profiling, and 28 bodies were identified. Personal effects, clothing, medical devices, and a strong match between non-genetic AM and PM data led to an attribution of identity of other 19 individuals. Advanced post-mortem phenomena were observed in remains spanning from the late 19th century to 2017. An identification area at the cemetery facilitated streamlined operations, emphasizing environmental preservation and forensic accuracy. Conclusions: The cemetery collapse highlights the necessity for tailored forensic approaches in disaster scenarios. Accurate identification methods, combining genetic analysis and secondary means, are crucial for ensuring dignified burials and providing closure to affected families.

## 1. Introduction

In the field of forensic medicine, identification is the scientific process that lets one ascertain the identity of a person, specifically the recognition and demonstration of individual characteristics that unmistakably differentiate one person from another [1]. Identification is achieved by comparing known characteristics of an individual with those observed on the body of the subject to be identified [2].

The identification of human remains is a complex procedure, especially in cases involving multiple fatalities, such as mass disasters. The process becomes even more challenging when dealing with skeletal remains. In these cases, forensic anthropologists play a critical role in creating biological profiles, which are fundamental for finding correct matches but, however, cannot be used for identification purposes alone [3,4,5].

The complexity of identification efforts necessitates a multidisciplinary approach that is both standardized and adaptable to the specific context. To this end, Interpol developed the Disaster Victim Identification (DVI) protocol, which comprises four phases: sample collection, post-mortem data collection (PM), ante-mortem data collection (AM), and the comparison of AM and PM data [6].

This protocol has proven effective in various mass disaster scenarios and is the standard applied also in Italy [6,7,8,9,10]. However, international identification protocols are more readily implemented in scenarios where data collection is relatively straightforward (e.g., when victim identities are known, close relatives are easily traceable, and identifications can be performed within a limited timeframe) [7]. Challenges arise in cases with significant obstacles to data collection, such as difficulty in locating close relatives, long intervals since the last contact with the victim, or a lack of usable material for identification purposes (e.g., suitable photographs, dental, or medical records). These issues represent not only scientific challenges, as they hinder the identification of all individuals, but also organizational challenges due to the need for adequate facilities, time, personnel, and financial resources, which are not always available [2,5,11].

Moreover, unlike other countries, Italy has historically overlooked the role of forensic medicine in mass disaster scenarios requiring the identification of multiple victims. Only in recent years, thanks to the efforts of numerous forensic professionals, has there been increased legislative interest. Forensic medicine plays a fundamental role in mass disasters, addressing both medical aspects (e.g., identifying bodies and determining the causes of death) and institutional aspects (e.g., liaising with judicial and health authorities). The inadequate inclusion of forensic medicine in national disaster response plans remains a significant issue, negatively impacting emergency management and outcomes [3,4,5,12].

These arguments were highlighted in the event under study, represented by the collapse of a coastal cemetery [13]. This context has created a unique situation requiring the identification of a large number of human remains in highly variable post-mortem conditions, with some deaths occurring several decades prior. The circumstantial reconstruction of the collapse was made possible thanks to newspapers articles, the information provided by the local Municipality and the Law Enforcement involved in the recovery operations: on 22 February 2021, the oldest part of the Camogli cemetery, in the province of Genoa (Italy), collapsed into the sea due to a rockslide. The coastal erosion caused the failure of the structure located on a sea cliff, scattering hundreds of corpses and skeletal remains.

The identification of the remains from the collapse was essential to uphold the dignity, honor, reputation, history, identity, and cultural heritage of the deceased, as well as the rights of their families [14,15,16,17]. This process helped prevent families from experiencing a “secondary” loss, characterized by uncertainty about the location of their loved ones and the absence of a site to honor them [18]. Additionally, the identification efforts had legal implications, including the need to exclude non-cemetery-origin remains and enable families to pursue legal actions against those allegedly responsible for the event. Amidst the complex management, during the first weeks after the events, there was a long period of turmoil in which LABANOF (the Forensic Anthropology and Odontology Laboratory of Milan, specializing in the identification of human remains also in complex scenarios) had been consulted by both authorities and families of the presumed decedents involved. LABANOF referred authorities to the Institute of Legal Medicine of Genova, providing only initial support from the forensic anthropological perspective. The identification procedures were subsequently assigned to the Legal Medicine Section of the IRCCS Policlinico San Martino Hospital of Genoa. LABANOF, hence, assisted only in the preparation of forms and in the initial preparation of the anthropological work setting. In this period, an attempt was even made to incorporate into the workflow standardized X-rays of the human remains to the fullest extent through a “Rextar-X” portable X-ray unit (Posidion Co., Ltd., Seoul, Republic of Korea) and an X-DR L Wi-Fi detector (Examion ©, Fellbach, Germany). This, however, did not comply to the logistics of the situation and was not continued.

This study aims to describe and evaluate the forensic activities conducted following the collapse of the Camogli cemetery in light of the existing literature and Italian legal standards. Strengths and numerous challenges encountered during the process are analyzed and shared to draw attention to the difficulties of similar situations and underscore the need for improved national and institutional operational protocols.

## 2. Materials and Methods

### 2.1. Human Remains Recovery

The landslide event resulted in the dispersion of 370 deceased individuals, more than 200 of whom reached seawater below [13]. The remaining ones were confined to the terrestrial portion of the landslide. Recovery operations were carried out with the support of firefighters and scuba divers. These complex recovery efforts involved both the affected section of the sea and the landslide-affected terrain. The operations lasted for over a month following the collapse.

Many of the remains involved in the landslide were only partially recovered, and some were never found. Given the collapse into the sea, the action of marine currents, and the significant risks associated with recovering remains from beneath an unstable landslide, it was not feasible to retrieve all skeletal elements. Additionally, environmental factors, including marine fauna and scavenging birds observed over the site, further complicated the preservation and recovery of remains.

The recovered bodies and remains were temporarily placed in a protected area of the cemetery.

The Public Prosecutor’s Office initially opened a case for the offense of environmental disaster but subsequently deemed it unnecessary to include identification efforts in the investigation. Nevertheless, given the importance of returning the remains to the families and regardless of the judicial requirements, identification efforts were pursued. In May 2021, the Legal Medicine Section of the IRCCS Policlinico San Martino Hospital of Genoa stipulated an agreement with the Municipality of Camogli to identify the remains, comprising 140 bodies and 193 bags of commingled skeletal remains. Not all 370 deceased individuals involved in the collapse required identification, as 37 intact coffins and urns bearing nameplates were deemed already identified.

### 2.2. Identification Protocol

Previous broad identification projects [19] highlighted the necessity of standardized protocols during the corpse recognition phase in mass disasters. In the present case, given the post-mortem cadaveric dispersion and the need to simultaneously process multiple bodies or their remains, a specific identification protocol was developed based on the Interpol DVI model [20] due to the unique characteristics of the specific case. The key stages of this adapted operational protocol can be summarized into four phases: (I) disaster scene analysis and operational planning; (II) compilation of post-mortem forms (PM) by the forensic team, including information obtained from the examination of bodies and remains; (III) compilation of relatives information form by relatives (a modified version of the AM form, incorporating information regarding the circumstances of death, burial-specific details, such as clothing or objects placed in the coffin, and information about kinship relationships); (IV) comparison of databases generated drawing data from the PM and relatives information forms. Finally, genetic investigations were carried out for identification purposes.

#### 2.2.1. Operational Planning (I)

The Municipality of Camogli provided a list of the remains found during the recovery operations to which an identification number had already been assigned along with the sector in which they were located. The first site inspection was carried out at the cemetery to assess the condition and the number of bodies and remains present, as well as to set up the workstation dedicated to their study. Work kits for the inspection were also prepared, including various copies of PM forms, personal protective equipment for health operators, identification tags and a permanent marker, measuring tape for the bodies or bones, and specific equipment for collecting dental, bone, or other sample matrices (labels, test tubes, swabs, bags for biological material, scalpels, forceps, pliers, saws, and sample containers). In order to prevent contamination for potential genetic analyses, sodium hypochlorite and a dedicated container for the instruments’ decontamination were also included in the work kits.

At the same time, the identification and training of forensic medical personnel with experience in various forensic fields were carried out, organizing teams composed of at least two forensic pathologists and one technical assistant.

#### 2.2.2. Post-Mortem Forms (PM) (II)

The PM forms were devised following the guidelines of the DVI [20], aimed at obtaining information regarding specific physical traits of the deceased. In addition, the biological profile for every case was created for narrowing down possible identities. The consequent anthropological analysis was performed to assess three parameters: sex, age, and stature. The form included the following fields:

- Identification code;
- Identification bracelet;
- Sex;
- Estimated age;
- Height;
- The condition of preservation;
- Clothing;
- Jewelry and objects such as rings, piercings, watches, other jewelry, and any items found in the coffin, with particular attention to personalized objects;
- Hair (present/absent–short/long), mustache, and beard;
- Scars, evident deformities, facial features, and distinguishing marks;
- Tattoos;
- Prostheses and devices: pacemakers, dentures and implants, orthopedic prostheses, cosmetic prostheses, drug injection pumps, or others;
- Signs of ante-mortem trauma;
- Autopsy/diagnostic post-mortem examinations/multi-organ sampling;
- Dental conditions;
- Samples collected;
- Work team indication and date.

The technical procedures were carried out by individually examining each body or group of remains, following the standard procedure outlined in the PM forms and using the equipment described. First, it was verified that the number assigned to the material under study appeared on the list of recovered remains provided by the Municipality of Camogli. The same number was then placed on the PM form and written on a specific identification tag used for photographs and any objects or clothing found together. The actual post-mortem examination was conducted by performing a general assessment of the body or remains, paying attention to the possible presence of autopsy traces, prior interventions, or conditions useful for identification, followed by forensic anthropological analysis as indicated in the literature [21]. Dental elements and samples from long bones were collected for possible DNA analysis. Each sample was stored in a dedicated biohazard plastic bag, sealed with a tag bearing the unique identification number, as indicated by national and international guidelines [22,23,24]. Regarding the biological profile, sex was inferred from the external appearance for the bodies in good conditions. In other cases, morphological and metric features of the skull and pelvis were evaluated (e.g., the evaluation of the Phenice triad) [25,26]. The assessment was based on the greatest compatibility of the traits with one of the two sexes. Age, in well-preserved bodies, was also inferred from the external appearance, while in the remaining cases, the methods of Suchey-Brooks, Meindl, Lovejoy, and Iscan were combined [27,28,29]. Finally, stature was assessed through direct measurement of the body, if intact, or calculated using osteometric tables based on the length of the femur and/or humerus [30]. For sex, age, and stature, if the collected data were insufficient to provide a reliable judgment, the parameter was considered not evaluable.

In the case of commingled remains, after evaluating the ethical, administrative, and legal usefulness of identifying individual bone fragments within this specific context, only photographic documentation and compilation of the PM form were carried out. For this material, the minimum number of individuals was determined, without conducting sampling for genetic identification and omitting the assessment of biological profiles.

A total of 21 cemetery accesses were carried out from May to August 2021. The compiled PM forms were entered into a dedicated Microsoft Excel database for subsequent comparison with the ante-mortem forms.

#### 2.2.3. The Modified Ante-Mortem (AM) Forms, Best Indicated as Family Data Collection Forms (III)

The family data collection forms, developed specifically for this event, were distributed to relatives and subsequently completed by them. The compilation involved the inclusion of information typically requested in DVI ante-mortem forms [20], along with additional details concerning burial and the kinship of the deceased individual. These forms, despite originating from standard AM forms, differ from the latter as the requested information concern individuals whose death was already known and certified by the competent authorities. Like PM forms, the modified AM forms were also entered into a dedicated Microsoft Excel database.

The form was structured as follows:

- Personal information regarding both the deceased and the form compiler, including their degree of kinship. This was intended to facilitate recontacting the relative if further information was needed and to reconstruct genealogical trees to plan potential genetic investigations. For the same purpose, relatives were asked to provide information on other close family members, who could serve as sources for DNA sample collection;
- Information on physical characteristics, including medical data. This encompassed any previous surgeries, illnesses, and details regarding dental condition. Additionally, respondents were asked to indicate whether an autopsy had been performed on the body;
- Burial information, including details about the clothing worn by the deceased at the time of burial and the presence of any specific items placed in the coffin;
- Photographs and available radiographic examinations, including dental images.

#### 2.2.4. Relatives’ Information and PM Database Comparison (IV)

The data obtained from the collection of relatives information form and PM data, entered in their respective databases, were compared for the identification purposes. Possible matches were manually highlighted by forensic personnel. When one or more distinctive elements were identified in the PM database, the same elements were searched for in the relative information form database. Given the specific circumstances, considering the limitations arising from the context and the absence of a dedicated authority in charge for deploying all resources for a scientifically robust anthropological identification, the remains were returned to relatives based on high compatibility between the information provided by the families and the PM data, along with the presence of burial-related features and markers.

### 2.3. Genetic Analysis

In cases where secondary identification techniques did not yield positive results, genetic profiling analyses were applied as a primary identification method [31]. In the present context, this method enabled the highest number of identifications through the analysis of short tandem repeat (STR) polymorphisms. The genetic analyses were organized into three phases: collection from the corpses or remains, collection from family members, and comparison of the obtained genetic data.

#### 2.3.1. Sampling from Corpses or Remains

During the accesses to the cemetery, dental elements were collected from the corpses when possible. Alternately, a portion of the diaphysis was sampled from the femora and humeri. All the samples were labeled with the corpse’s identification number and stored in refrigerated cells. As explained above, because of the limited resources and the institutions involved, it was decided not to proceed with DNA analyses in cases of avulsed teeth or commingled remains where it was not possible to discern the bones belonging to specific individuals. Isolated teeth that were not clearly associated with a specific skull were not analyzed. Similarly, commingled remains were not sampled for analysis unless it was possible to reconstruct and return most of an individual’s body to their family.

Given the mineral nature of the biological material being studied, the first procedure performed was the decalcifying pretreatment. Before this step, any soft tissue was removed using disposable scalpel blades, without the use of any chemical agent. The exposed external surface of the dental or bone sample was decontaminated by sequentially using sodium hypochlorite 4%, absolute ethanol, and ultrapure water. Subsequently, the diaphyseal portion of the long bones were longitudinally sectioned. One gram of the produced bone powder or scales was carefully collected in a decontaminated tube and pulverized in a warring mill according to validated protocols [32]. Each processed bone material was placed into a 50 mL tube, labeled in accordance with the corpse or remains from which they were obtained. For the teeth, these were individually wrapped in DNA-free cotton bands and placed inside a biohazard sampling bag. Then, they were manually crushed with a hammer on a surface previously decontaminated with diluted sodium hypochlorite. The produced fragments were placed in labeled 50 mL tubes.

Demineralization was carried out by adding 8 mL of 0.5 M EDTA at pH 8 to each sample tube. After vortex homogenization, the tubes were placed in an agitator for 24 h at room temperature to facilitate the removal of the mineral matrix. The tubes were then centrifuged at 3000 RPM for 30 min, the liquid waste was discarded, and the sedimented bone residues were retained. The EDTA treatment was repeated in the same manner for a second time. The organic sediment, once again deprived of the waste supernatant, was subjected to cell lysis. Lysis was performed slightly adapting the Robino et al. protocol [33] by adding 7.5 mL of a solution consisting of 50 mL proteinase K at 20 mg/mL, 375 mL Tris-HCl 1 M at pH 8, 375 mL SDS 10%, and 6.7 mL EDTA 0.5 M at pH 8. The tubes were then placed on a vortex and subsequently in an agitator for 48 h at 40 °C. DNA extraction was carried out from the resulting mixture using the QIAamp^®^ DNA Blood Maxi Kit (QIAGEN^®^, Hilden, Germany) following the “QIAamp^®^ Blood Maxi Kit Spin Protocol” [34].

The obtained nucleic acid extract was further purified using the QIAamp^®^ DNA Investigator Kit (QIAGEN^®^, Hilden, Germany) following the “Isolation of Total DNA from Tissues” protocol, with appropriate adaptations as follows:

- To the previously obtained solution, add 1 mL of Buffer AL and mix thoroughly vortexing before placing it in a thermomixer at 56 °C for 10 min.
- Add 1 mL of 96–100% ethanol and vortexing.
- Transfer the mixture into the QIAamp^®^ MinElute Column (QIAGEN^®^, Hilden, Germany) in 5–6 steps of 500 mL each as the maximum volume of the column is 500–700 mL. After each step, centrifuge at 14,000 RPM for 1 min, replacing the collection tube and discarding it along with the corresponding filtrate.
- Add 500 mL of Buffer AW1 and centrifuge at 8000 RPM for 1 min.
- After changing the collection tube, add 700 mL of Buffer AW2 and centrifuge at 8000 RPM for 1 min.
- Replace the collection tube and add 700 mL of 96–100% ethanol.
- Using a new collection tube, dry the column by centrifugation at 14,000 RPM for 3 min.
- Transfer the column into a sterile 1.5 mL tube and add 35 mL of Buffer ATE, incubating for 5 min at room temperature before centrifuging at 14,000 RPM for 1 min.
- Discard the column and retain the tube containing the DNA extract.

As justified in the discussion and given the advanced state of decomposition of the corpses or remains involved in the cemetery collapse, the nucleic acid quantification was bypassed, employing pure DNA extracts for the amplification step. The amplification of STR markers was performed using the PowerPlex^®^ Fusion System (Promega Corporation, Madison, WI, USA), followed, when necessary, by the PowerPlex^®^ 16 HS System (Promega Corporation, Madison, WI, USA). Positive and negative PCR controls were used to monitor contamination. Whenever the use of the first kit alone produced electropherograms that were highly incomplete or difficult to interpret, the second kit was subsequently applied to the same sample. In such cases, compositum STR profiles were generated according to the literature [35]. When subjects were genetically identified as male, Y-chromosome polymorphism study was also applied, performing the amplification with the PowerPlex^®^ Y23 System (Promega Corporation, Madison, WI, USA).

The analysis of the amplified STR fragments was carried out using the ABI PRISM^®^ 310 Genetic Analyzer (Thermo Fisher Scientific™, Waltham, MA, USA). Adopting a 47 cm capillary and POP-4 polymer, the instrumental parameters were set as follows: injection time at 5 s, injection voltage at 15.0 kV, run voltage at 15.0 kV, run temperature at 60 °C, and run time at 28 min. It is noteworthy that the genetic investigations were conducted during 2021 and 2022 when the instrument was still maintainable. The allele calling was made using GeneMapper ID v3.2 software (Thermo Fisher Scientific™, Waltham, MA, USA), setting the analytical threshold at 100 RFU. The resulting genetic profiles were entered into a Microsoft Excel database dedicated to unidentified remains.

#### 2.3.2. Sampling from Relatives

In order to proceed with genetic comparison, it was first necessary to identify suitable relatives for the analysis. This selection was performed following manual reconstruction of genealogical trees, which were generated based on the data provided by the relatives themselves. Whenever possible, first-degree relatives were selected as each parent contributes 50% of an individual’s nuclear genetic material. Additionally, for male relatives who had a male child or brother available, Y-chromosome analysis was carried out to enhance the discriminating power.

In certain selected cases, second-degree relatives were also considered, particularly when both autosomal markers and Y-chromosome data could be obtained or when a parental gap could be bridged by identifying a connecting relative. For example, if a man was identified using secondary identification techniques, but his father and brother were not yet identified, the DNA of the identified individual’s male child was required to provide a less degraded DNA sample for comparison.

The relatives DNA was collected using a buccal swab. Prior to the procedure, the methods and purposes of the sample collection were thoroughly explained to the probands. Informed consent forms were completed and signed. The swabs were subjected to DNA extraction using the Gentra^®^ Puregene^®^ Blood Kit (QIAGEN^®^, Hilden, Germany), following the protocol for “DNA Purification from a Buccal Brush”.

Amplification was carried out using the PowerPlex^®^ Fusion System (Promega Corporation, Madison, WI, USA), while Y-chromosome polymorphisms were analyzed with the PowerPlex^®^ Y23 System (Promega Corporation, Madison, WI, USA).

Analysis of the amplified STR fragments was conducted using the same instruments and software previously described for obtaining genetic profiles of cadavers or remains. The analytical threshold was set at 150 RFU. The resulting profiles were entered into a Microsoft Excel database dedicated to relatives of the missing cadavers.

#### 2.3.3. Genetic Data Comparison

Genetic profiles obtained from unidentified cadavers and their relatives were manually compared by evaluating the alleles shared for each STR marker until achieving a familial match based on the observed and reported degree of kinship. In detail, the number of autosomal markers that have at least one allele in common between two profiles was checked. Based on the mendelian inheritance laws, considering the 22 autosomal target loci of the PowerPlex^®^ Fusion System and the 15 autosomal target loci of the PowerPlex^®^ 16 HS System, the minimum number of STR markers required to establish the kinship was set at 18 out of 22, either for compositum profiles and single amplification profiles. This threshold was determined considering first-degree relatives, the individual mutation rate, and the potential presence of uninformative loci derived from low DNA quality or quantity. For the few specifically selected second-degree relatives, the minimum number of STR markers was set at 11, integrating these data with the compatibility of Y-chromosome polymorphisms, and the ante-mortem records, and considering the kinship degree declared by the relative truthful.

To avert false attributions or exclusions of kinship, genetic profiles generated from the analysis of electropherograms with particularly challenging interpretations or associated with the simultaneous presence of multiple contributors were deemed unsuitable for database comparisons.

The Microsoft Excel files containing the alleles detected during genetic analysis were processed for statistical kinship analysis. Genetic profiles were subjected to probabilistic analysis using dedicated software, Familias V. 3.1, setting the posterior probability for a positive identification to more than 0.999 [31,36,37] and assuming a uniform prior.

Once the statistical results were obtained, an additional manual verification was performed, cross-referencing the post-mortem findings with the corresponding profile identified as a relative and the data collected from family members. This step ensured that no anthropological inconsistencies were present in the available information.

## 3. Results

A total of 333 post-mortem records were compiled, consisting of 140 bodies, 163 skeletal remains, and 30 commingled remains. Concerning the sex, the analysis of biological profiles allowed the identification of the following:

- 136 males;
- 114 females;
- 53 of undeterminable sex;
- 30 unassessed, corresponding to the commingled remains.

Regarding the age, the following was noted:

- 163 individuals aged 65 years or older;
- 87 individuals aged between 20 and 65 years;
- 1 subadult aged between 3 and 5 years;
- 52 were of an undeterminable age;
- 30 unassessed, corresponding to the commingled remains.

In 129 cases, sex and age were assessed based on external appearance, given the limited decomposition of the cadavers. The remaining cases did not allow for the determination of sex and age due to the limited number of recovered bones available for analysis.

For the remaining cases, the anthropological or genetic techniques previously described were applied.

Concerning the data collection forms obtained from family members, a total of 154 modified AM forms were fulfilled, pertaining to the following:

- 76 males and 78 females;
- 123 individuals aged 65 years or older, 30 individuals aged between 20 and 65 years, and 1 individual aged 13 years.

Regarding medical information, particularly radiographs and dental data, no ante-mortem orthopantomogram or radiographic images were provided. However, medical and dental generic information (e.g., the presence of full dentures, either upper or lower) was available in approximately 75% of cases (115 cases). In no instances were identification codes for prosthetics or pacemakers provided.

For 19 cadavers in which genetic identification was not possible but AM and PM data provided a strong match based on personal descriptors, it was decided that the additional presence of distinctive burial elements could allow for a name to be attributed to the body regardless of the lack of a scientific method, which could prove per se identity beyond reasonable doubt. In absence of judicial authority involvement, this decision was taken in agreement with the municipality and the relatives. A summary of the cited elements used for each of these 19 individuals is presented below (Table 1):

For all the other corpses or remains, the genetic identification was pursued. Once the familial compatibility between relatives and the missing deceased was assessed as described earlier, out of 154 bodies for which identification was requested, 77 relatives were deemed suitable for 71 of the bodies. A total of 66 relatives, corresponding to 69 bodies, provided their consent for the collection of biological samples and subsequent typing.

Regarding the missing deceased, among the 333 post-mortem forms potentially corresponding to 333 identifications, certain categories of remains were deemed unsuitable for comparison. These included commingled remains, remains containing only a minor portion of the skeletal system, those in an advanced stage of decomposition, ashes contained in urns of cremated individuals, and samples with ambiguous individuality, even if not overtly commingled. All such cases were considered unreliable for identification.

Following this selection process, a total of 139 bone samples were collected, including 66 long bones and 73 teeth, corresponding to 125 bodies. Of these, excluding the deceased identified using secondary identification techniques, 115 samples required genetic analysis. However, 42 of these samples were found to be highly degraded, particularly difficult to interpret or associated with multiple contributors, making them unsuitable for comparison with relatives. For other samples, all negative PCR controls showed no signal of amplification or allele calling, and positive PCR controls were correctly typed as indicated in the corresponding amplification kit. Forty-five samples were deemed suitable but could not be attributed, while the identification of 28 bodies was successfully achieved through genetic methodologies. The corresponding posterior probabilities were greater than 99.9% in all cases.

For each of these cases, additional verification was performed using the corresponding ante-mortem records, confirming compatibility in all instances.

In summary, out of 154 bodies for which families requested identification, considering that only 69 allowed for a suitable genetic comparison, the identification of 47 bodies was achieved: 19 of which through secondary techniques and 28 through primary techniques. For each identification, a formal notification was sent to the Italian Local Health Authority (Autorità Sanitaria Locale—ASL), which included the recognized individual’s details, the identification number assigned to the body, and the detailed rationale behind the identification. The ASL subsequently proceeded with the completion of the final identification document, which was forwarded to the Municipality of Camogli to facilitate legal procedures and burial or cremation.

## 4. Discussion

Forensic medicine plays a pivotal role in events involving large numbers of individuals. Its dual purpose includes medical objectives, such as assigning proper identities to victims and determining causes of death, and legal responsibilities, acting as an intermediary between the judicial system and the medical sector [11,16].

The complexities of forensic activities in mass disasters stem from several intrinsic factors, which are often exacerbated in large-scale events. These include the high number of individuals involved, variability in the condition of bodies and their progressive decomposition (frequently resulting in the discovery of only skeletal remains), unfavorable environmental and topographical conditions, media and official pressures, limited time to organize and manage activities (due to the urgent need for rapid response), the emotional resonance of the event, and the involvement of numerous professionals and non-professionals who may interfere with forensic activities, including bereaved families requiring attention from healthcare professionals [36,37,38,39,40,41].

As previously discussed, the role of forensic medicine in mass disasters is, therefore, crucial yet highly complex, requiring adequately trained personnel and the application of standardized procedures. While the Interpol DVI protocol is widely and effectively applied, the national literature highlights cases where adaptations may be necessary. These adaptations are particularly relevant in scenarios where challenges arise in the collection of ante-mortem (AM) and post-mortem (PM) data [39,40,41,42].

Scientific and organizational models employed by individual national teams in various mass disasters have been well documented in the literature. For instance, Cattaneo et al. [11,42], in their account of the 2001 Linate airplane disaster, emphasized the need for forensic medical activities to follow distinct phases. Cecchi et al. [43] focused on identification procedures, describing approaches used by the University of Rome “La Sapienza” in mass disasters occurring between 1964 and 2005. They both underscored the importance of forming a multidisciplinary team of forensic experts, such as pathologists, odontologists, geneticists, radiologists, fingerprint experts, photographers, data recorders, autopsy technicians, psychologists, law enforcement personnel, and support staff. In addition, in their work on identifying deceased Mediterranean migrants, Cattaneo et al. [39] noted that in certain circumstances, so-called secondary identification methods can achieve identification if appropriate AM and PM data are available and if a rigorous comparative statistical procedure is applied. This observation is corroborated by a recent position statement by the Forensic Anthropology Society of Europe [44], which demonstrated that anthropological identification methods can be effectively employed to help achieve individual identification.

Regardless of the models proposed, various authors consistently highlight the need to develop adaptable scientific and organizational protocols that involve forensic medicine to address mass disasters effectively [45,46,47,48,49,50,51,52,53,54,55,56].

In the presented case, the collapse of the Camogli cemetery in February 2021, resulting in the dispersal of numerous bodies into the sea, represents a complex and unique case where forensic medicine played a decisive role in organizing and implementing identification procedures.

The identification of the bodies involved in the collapse was essential to ensure, on the one hand, respect for the dignity, honor, reputation, history, identity, and culture of the deceased—rights that are legally protected—and, on the other hand, the rights of the families. Identification prevented families from experiencing a “secondary” loss, marked by uncertainty over the location of their loved ones and the absence of a site for mourning and remembrance. Additionally, in this case, identification carried legal significance, both criminal and civil, to rule out the possibility of remains unrelated to the cemetery and to enable families to pursue legal actions against those allegedly responsible for the collapse.

From the outset, numerous operational challenges became apparent.

Common to all events involving large numbers of individuals were issues such as the lack of shared scientific and organizational strategies for cases with significant data collection difficulties; the imbalance between the high number of bodies and the limited resources available, encompassing economic resources, appropriate facilities for large-scale identification operations, and personnel with specialized skills; the time factor, with rapid progress demanded by authorities and necessitated by decomposition processes and the need to free up space, versus the lengthy procedures required for proper identification under limited resources; and the pressure from media and authorities, alongside the emotional resonance of the event, with numerous operators and non-specialists (journalists, victim associations, etc.) potentially interfering with medico-legal activities.

Beyond these common challenges, the collapse of the cemetery also presented specific issues. Chief among them was the absence of similar precedents in the literature, which primarily details cases of mass disasters involving recent victims or archaeological/scientific studies of cemetery remains [57,58,59]. This cemetery collapse represented a scenario intermediate to these two situations. Further complications included the lack of forensic involvement in body recovery operations, resulting in the inadequate application of DVI protocols, frequent commingling of remains, and an inability to trace precise recovery locations; the compromised and variable conditions of the remains due to post-mortem changes, their fall, and subsequent time in seawater; inaccuracies in cemetery records provided by the municipality, including duplicate names and nicknames, partly because the collapsed section was the oldest part of the cemetery; the long post-mortem intervals of the remains, ranging between the end of the 19th century and 2017, which posed challenges in collecting data from relatives and in genetic analysis. Finally, the operations were financed by the municipality of Camogli, which, as the commissioning entity, sought active participation in organizing the procedures, sometimes posing challenges to forensic operations.

In response to these myriad challenges, the Legal Medicine Section of the IRCCS Policlinico San Martino Hospital of Genoa developed a specific operational protocol based on Interpol’s DVI guidelines. While not intended to be universally applicable, this protocol achieved positive results and may provide insights for future strategies. The protocol’s key steps can be summarized as follows:

1. Scene Analysis and Operations Planning: Inspections were conducted, and work kits prepared to ensure suitable identification stations.
2. Post-mortem Data Collection: Forensic pathologists completed PM forms based on cadaver examination findings.
3. Family Data Collection Forms: Relatives filled out the modified AM forms, providing pre-death information about their loved ones.
4. Data Comparison and Genetic Testing: Databases created from PM and AM data were compared, with genetic testing as a means of scientific positive identification.

The preference for genetic methods over other primary identification techniques was dictated by the case’s specifics, including the quality of data available from both cadavers and relatives and the scarcity of resources. However, even the genetic analyses presented several critical challenges. The extended period elapsed between death and recovery resulted in difficulties in the identification of living relatives suitable for genetic comparison. Additionally, close intrafamilial relationships, both among the missing individuals and the identification claimants, further complicated the genetic investigations. Finally, the time of death and the taphonomic conditions of the bodies or remains often resulted in advanced stages of decomposition, directly associated with macroscopic and molecular degeneration [60,61]. The consequences are evident in many of the obtained genetic profiles, which can be difficult to interpret or uninformative due to the presence of degraded DNA, reaction inhibitors, or scarce quantities of nucleic acids [62,63,64]. This partly could explain the failure to genetically identify most cadavers or remains among those subjected to analysis.

Although this departure from DVI protocol may appear unconventional, every identification scenario requires specific adaptations: in this case, decisions were made to maximize identification potential given the case’s unique characteristics. Innovations introduced by the protocol included the following:

- *Operational Organization:* Inspections and preparation of identification stations, as well as the selection and training of healthcare personnel, resulted in the creation of multidisciplinary forensic teams specializing in genetics, anthropology, entomology, pathology, and odontology.
- *Adjustments to Identification Protocols:* Unlike DVI’s extensive use of primary identification techniques, there were cases in which genetics was not utilized. Given the limited resources, genetic analysis was employed in cases when no other highly compatible elements were available. This decision was driven by the low quality of cadaveric material, affected by extended post-mortem intervals and exposure to water, and the need to allocate limited resources effectively. Additionally, AM forms were adapted into relative information forms, including burial-specific details (e.g., clothing or objects placed in the coffin) and information about kinship relationships, enabling genealogical tree construction essential for prioritizing genetic investigations. This led, in the lack of genetic identification, and in the lack of AM and PM data appropriate for anthropological or morphological identification alone, to the decision to return the remains to families where there was a match between AM and PM personal descriptors accompanied by distinctive burial features.
- *Communication Management:* Collaboration with multiple stakeholders (the Municipality of Camogli, local health authorities, and victim representatives) was fostered through shared planning during meetings, leading to an agreement between the IRCCS Policlinico San Martino Hospital and the Municipality of Camogli. Technological innovations, such as an explanatory video uploaded to the municipality’s website and a dedicated email address for inquiries and AM form submission, facilitated family participation while reducing travel and contact.

Despite significant challenges, these efforts yielded highly satisfactory results given the case’s complexity. Participation, measured by the number of completed family data collection forms, exceeded expectations, particularly since the collapsed section of the cemetery contained older burials, some over a century old.

The number of individuals identified was also commendable, with 47 identifications achieved—approximately one-third of cases submitted by families and about 75% of cases where primary identification techniques were applicable.

The operational protocol developed for this case demonstrates the value of targeted innovations and adjustments to address the specific challenges of complex identification scenarios.

## 5. Conclusions

As already introduced in the study on the preliminary identification activities conducted on the case of the Camogli cemetery collapse [13], the recognition of corpses and remains involved necessitated the development of a medico-legal operational protocol that addressed the absence of standardized procedures while tackling both the practical and logistical challenges. These challenges were partly common to all complex identification cases and partly specific to the unique circumstances of this event.

The multidisciplinary forensic approach developed and implemented during the identification procedures for the cemetery collapse into the sea represents a novel contribution to the field of disaster victim identification. Despite the significant operational challenges, including the complex recovery context, compromised condition of the remains, and limitations in resources, the tailored protocol proved effective in addressing case-specific needs while adhering to international standards. By integrating anthropological and genetic methodologies, along with innovative adaptations such as genealogical tree construction and enhanced communication strategies, this protocol underscores the importance of flexibility and inter-disciplinary collaboration in forensic operations. The outcomes, including the successful identification of a substantial proportion of individuals, highlight the relevance of adapting international guidelines like the DVI to local contexts. Although the need for a tailored approach is essential, in this case, the overall conditions did not allow for the achievement of the highest possible scientific standards. This was due to the absence of a dedicated authority responsible for such identifications, together with the time span of deaths, making it difficult to recover appropriate AM data from relatives, which, to this day, remain almost exclusively ethical endeavors in the absence of a structured and organized public framework.

This study not only emphasizes the critical role of forensic medicine in complex identifications but also advocates for the continuous evolution of national protocols and the establishment of dedicated multidisciplinary teams to address future mass casualty events effectively and to advance best practices in forensic identification under challenging circumstances. The flexibility and the establishment of structured organizations should not be seen as mutually exclusive; rather, they are complementary elements necessary to ensure the effective protection of rights with the support of science and appropriate technological innovations. Balancing these two dimensions is essential, particularly in forensic identification scenario, where the complexity of cases often demands both adaptability to unforeseen challenges and adherence to high scientific standards.

## Figures and Tables

**Table 1:** Strategies adopted for the 19 bodies returned to families without genetic analysis.

Number Assigned to the Corpse	Biological Profile (Sex, Age, Height, and Time of Death)	Connotations (Hair/Beard/Mustache, Physique, and Common Paraphysiological Conditions)	Consistent Personal Descriptors	Highly Distinctive Burial Elements
1	Compatible	Compatible	Scars, diseases	Clothes, items
2	Compatible	Compatible	Not present	Clothes, items, hospital ID bracelet
3	Compatible	Compatible	Tattoos	Clothes, items, hospital ID bracelet
4	Compatible	Compatible	Not present	Items, hospital ID bracelet
5	Compatible	Compatible	Not present	Clothes, hospital ID bracelet
6	Compatible	Compatible	Prosthesis	Clothes, hospital ID bracelet
7	Compatible	Compatible	Not present	Clothes, hospital ID bracelet
8	Compatible	Compatible	Not present	Items, hospital ID bracelet
9	Compatible	Compatible	Not present	Clothes, hospital ID bracelet
10	Compatible	Compatible	Not present	Clothes, hospital ID bracelet
11	Compatible	Compatible	Not present	Clothes, hospital ID bracelet
12	Compatible	Compatible	Not present	Clothes, hospital ID bracelet
13	Compatible	Compatible	Prothesis	Autopsy report, clothes
14	Compatible	Compatible	Not present	Clothes, hospital ID bracelet
15	Compatible	Compatible	Prosthesis, diseases	Clothes
16	Compatible	Compatible	Not present	Clothes, hospital ID bracelet
17	Compatible	Compatible	Not present	Clothes, hospital ID bracelet
18	Compatible	Compatible	Not present	Clothes, items
19	Compatible ^1^	Compatible	Diseases	Clothes, items

^1^ Subadult.

## Data Availability

All authors of the article allow to share the research data. Data will be provided upon reasonable request by the corresponding author.
